# Impact of Type of Parturition on Colostrum Microbiota Composition and Puppy Survival

**DOI:** 10.3390/ani11071897

**Published:** 2021-06-25

**Authors:** Leonida Kajdič, Tanja Plavec, Irena Zdovc, Anja Kalin, Maja Zakošek Pipan

**Affiliations:** 1 Krka d.d., Pharmaceutical Industry, 8000 Novo Mesto, Slovenia; leonida.kajdic@gmail.com; 2 Small Animal Veterinary Hospital Hofheim, Katharina-Kemmler-Straße 7, 65719 Hofheim, Germany; t.plavec@tierklinik-hofheim.de; 3 Institute of Microbiology and Parasitology, Veterinary Faculty, University of Ljubljana, Gerbičeva 60, 1000 Ljubljana, Slovenia; irena.zdovc@vf.uni-lj.si; 4 Small Animal Clinic, Veterinary Faculty, University of Ljubljana, Gerbičeva 60, 1000 Ljubljana, Slovenia; anja.kalin@gmail.com; 5 Clinic for Reproduction and Large Animals, Veterinary Faculty, University of Ljubljana, Gerbičeva 60, 1000 Ljubljana, Slovenia

**Keywords:** neonatology, birth, puppies, microbiota

## Abstract

**Simple Summary:**

It has long been believed that the bacteria present in milk and colostrum were due to contamination during suckling from the oral cavity of the newborn or the skin of the mother. Colostrum and meconium from newborns were considered sterile. In the last decade, human research has provided evidence that bacteria are present in colostrum, milk, placenta, and the intestine of the newborn. The colostrum microbiota appears to change greatly and very rapidly, and in humans it has been found that it can be influenced by the type of parturition. Because information on the colostrum microbiota in dogs is lacking, the objective of our study was to determine whether the type of parturition affects the colostrum microbiota and the growth and survival of puppies in early life. Bacteria isolated from maternal colostrum and puppies meconium were identified by mass spectrometry. The results of this study provide new information on the colostrum microbiome of healthy dams and suggest that the type of parturition influences the bacterial composition of the colostrum microbiota, which may be an important factor in weight gain and survival of puppies in early life.

**Abstract:**

The objective of our study was to determine whether the type of parturition affects the microbiota of the colostrum and the growth and survival of the puppies. Seventy-nine newborn puppies were divided into three groups regarding the type of parturition: vaginal delivery (VD), elective caesarean section (EL-CS), and emergency caesarean section (EM-CS). After the birth of the puppies, swabs of meconium were collected from the puppies and colostrum was obtained from the dam. Many aerobic and anaerobic bacteria were isolated and identified by mass spectrometry (MALDI-TOF MS). The colostrum microbiota of VD and EL-CS puppies contained a significantly higher abundance of bacteria belonging to the genera *Staphylococcus*, *Kocuria* and *Enterococcus* compared with EM-CS colostrum samples. The composition of the meconium microbiota of the puppies present at birth was similar to the colostrum microbiota of their mothers. It was also found that puppies without a meconium microbiota at birth gained weight more slowly compared with puppies with a meconium microbiota at birth. The type of parturition influenced the bacterial composition of the microbiota in the colostrum. Future studies are necessary to further define the significance of the observed differences in microbiota composition between EM-CS compared with EL-CS and VD colostrum microbiota.

## 1. Introduction

In mammals, colostrum is a beneficial, nutritious food that is critical for early neonatal survival [1]. It is produced in the mammary glands and may be present several days before or immediately after birth. Colostrum may persist until two days after birth. Survival of puppies is particularly dependent on colostrum during the first few weeks, as they are nearly agammaglobulinemic at birth. Only five to ten percent of immunoglobulins are able to cross the placenta. This occurs in the last third of gestation in the circumferential form with a hemochorial organization of the zonary placenta [2]. The uptake of IgG from the digestive tract occurs mainly in the first 12–16 h after birth, and the transfer of IgG from the digestive tract to the bloodstream is critical for infant survival [3]. In addition, colostrum in humans is an important source of 200 different species of bacteria, such as streptococci, lactobacilli and bifidobacteria, which have a pivotal role in the neonatal gut colonization and can affect the health and growth of infants, especially early in life. They are important for systemic and mucosal immunity to promote a strong and healthy gut microbiota. Natural antibodies and other active substances present in colostrum are important for supporting and maintaining an appropriate balance between healthy and potentially pathogenic gut bacteria [4]. Consequently, colostrum intake is not only necessary as a staple food and source of nutrients, but also promotes the development of an appropriate gut microbiome, preparing the newborn for life outside the uterus [5]. Although the specific vehicles involved in the formation of the colostrum microbiota are not yet known, there are some hypotheses. The first hypothesis is that they originate from the oral cavity of the newborn and through the skin surface around the mammary glands. The second is that the maternal intestinal bacteria are translocated by the dendritic cells through the intestinal epithelial barrier and transported to the mammary glands via the lymphatic circulation (an entero-mammary pathway) [6,7]. Recently, a human study indicated the possible role of the parturition type in the formation of the bacterial composition of human colostrum, as the concentration of different bacterial genera in the breast milk of mothers who had a vaginal delivery (VD) was higher than in those who had a cesarean section (CS) [8]. The same observations were confirmed in another human study in which the parturition type had a major influence on the composition of the microbiota in the colostrum [9]. Moreover, the gut microbiome of a newborn has been found to play an important role in maintaining health throughout life [1]. It reduces the risk of many diseases, i.e., gastrointestinal diseases, such as necrotizing enterocolitis, respiratory diseases, such as asthma and reduces inflammatory responses, thus preventing allergies [9,10,11]. It is well known that newborns gradually develop their own microbiota after birth and that many factors such as environment, diet and antibiotic intake influence its composition. However, in the first days of life, the composition of the microbiota in newborns consists mainly of the bacteria present in the colostrum [3,12]. This is important because in puppies, 75–90% of deaths occur in the first 2–3 days of life and sepsis is the most important cause of mortality [13,14].

The milk microbiota has been studied mainly in women [7,15] and in cows [16,17]. Some studies investigating the bacterial composition of the mature milk microbiota have also been conducted in other animal species such as mice, sheep, goats, donkeys, buffaloes, water deer and reindeer [18,19,20,21,22,23]. However, only few studies in humans and cattle have investigated the colostrum microbiota [9,24,25]. To the best of our knowledge, the colostrum microbiota in dams has not been studied yet. The objective of our study was to determine the presence of bacteria in the colostrum of dams and to determine whether the parturition type influences the colostrum microbiota and has an impact on the growth and survival of puppies in their early life.

## 2. Materials and Methods

### 2.1. Animals

The Ethics Committee of the Veterinary Faculty approved the study. Owners of the client-owned dogs signed a consent form allowing samples to be collected from the dam and the neonates. Detailed information on the dogs included in the study is presented in Table 1. All dams were fed only FDA approved food for pregnant dogs. Types of parturition were divided in three groups: the vaginal delivery (VD), the elective CS (EL-CS) and the emergency CS (EM-CS). Two to nine newborn puppies were delivered per parturition. The weight, a modified Apgar scale and presence of congenital malformations (i.e., oronasal fistula, cleft palate, number of toes, atresia ani) was noted at birth. Resuscitation of neonates was performed via ABC: rubbing to stimulate breathing, clearing mucus from the upper airways, oxygenation, warming, glucose delivery, and, in one case, external cardiac massage. Puppies were marked with a colored collar immediately after birth to allow for the proper identification of puppies and collected samples.

### 2.2. Cesarean Section (CS)

When a medical management of dystocia failed or was inadvisable, EM-CS was performed. The day of the surgical delivery in EL-CS cases was determined by using the guidelines for an average gestation length of 63 days after the ovulation, coupled with the information gathered by serial monitoring of the blood progesterone concentrations and fetal ultrasonographic measurements of the internal chorionic cavity and biparietal diameter. The general health status of the dam was assessed by the clinical examination, complete blood count (ADVIA^®^ 120, Siemens; Munich, Germany) and venous blood gas analysis (VetScan i-STAT^®^ 1, Abaxis; Union City, CA, USA). The cephalic vein was cannulated for intravenous fluid and drug administration and 10 min long preoxygenation with 100% oxygen was used before the induction of anesthesia. The abdomen was shaved and washed with mild soap (Aniosgel 85 NPC, Ecolab Deutschland GmbH, Monheim am Rhein, Germany), rinsed with water, dried, and finally disinfected with propan-2-ol and benzalkonium chloride (Cutasept F, Bode, Germany) first with a spray and three minutes later with the paint technique. The anesthesia induction was performed by IV application of propofol (4–7 mg/kg body weight (BW)) (Propoven; Fresenius Kabi Ltd.; Runcorn; UK) followed by an orotracheal intubation. General anesthesia was maintained with sevoflurane (Sevofluran; Draeger; Luebeck; Germany) at a concentration of 1.5–3%. Methadone (Comfortan; Dechra; Northwich; UK) at a dose of 0.2 mg/kg BW was administered subcutaneously after the skin incision. A balanced isotonic crystalloid solution (Hartmann solution; B. Braun; Melsungen; Germany) was administered intravenously at a rate of 5–10 mL/kg/hour during the procedure. CS was performed with a caudal midline abdominal incision. Once the uterus was exposed, a ventral incision was made in the uterine body. The fetus that was the most caudal was removed first with its fetal membranes, followed by the fetuses and fetal membranes from, alternately, the right and left uterine horns.

To promote uterine contractions and cleansing, 0.25–1 IE oxytocin (Oxytocin; veyx-Pharma Gmbh; Schwarzenborn; Germany) was injected into the uterine wall. Before the routine abdominal wall was closed in a routine manner, the abdominal cavity was irrigated with 0.9% sodium chloride (NaCl; B Braun; Melsungen, Germany) warmed to body temperature.

Methadone at a dose of 0.2 mg/kg BW given subcutaneously was repeated four hours after the first dose, after which the dam and her puppies were discharged from the hospital. Tramadol chloride (Tramal; Stada; Bad Vilbel; Germany) at a dose of 3 mg/kg BW orally every 12 h was used for postoperative analgesia, as needed, for a maximum of three days.

### 2.3. Vaginal Delivery (VD)

Puppies born vaginally were delivered in the home of the owners.

### 2.4. Collection of the Colostrum

Before the collection of the colostrum, the nipples were disinfected with propan-2-ol and benzalkonium chloride. Samples were collected from the caudal pair of the mammary gland by milk expression while using sterile gloves. The first few drops of colostrum were discharged.

In the VD group, colostrum was expressed right after the birth of the first puppy and again four hours later. In the EL-CS and EM-CS groups, colostrum was expressed right after the surgery and again four hours later.

The puppies were allowed to suckle again after sampling and cleaning the mammary gland with sterile Ringer’s lactate.

### 2.5. Collection of the Meconium

Immediately after the first colostrum intake, the collection of the meconium was performed. For this purpose, sterile cotton swabs were soaked in warm sterile water. Using a circular massage around the perineal area of the newborn puppies, the expression of the meconium was stimulated. Dams were not allowed to lick their puppies before the collection of the meconium to prevent contamination of samples with the oral microflora from their mothers. The collection of the meconium was performed immediately after expression of the meconium with sterile swab.

### 2.6. Monitoring Puppy’s Growth

The weight gain of puppies was monitored until they reached eight weeks. Delivery day was marked as day 0. During the first three weeks, puppies were weighed every morning. Later, the body weight was measured once a week at an interval of 7 days.

### 2.7. Bacteriological Examination

Samples for bacteriological examination were taken from bitches and their puppies with sterile swabs and directly inoculated on blood agar plates (Blood agar base, Oxoid, UK, supplemented with 5% sheep blood). Each sample was examined regarding aerobic and anaerobic bacteria and therefore inoculated as duplicates—one of which was incubated under aerobic conditions and the other under anaerobic conditions created by the use of anaerobic atmosphere generating bags (GENbag, GENbox; bioMérieux, Inc.; Durham, NC, USA). The cultures were incubated at 37 °C for at least two days, anaerobic incubation was extended to 5 days. After the incubation period, the plates were checked for microbial growth and all distinctive morphological types of the colonies were subcultured onto fresh blood agar to obtain a pure culture. Subcultures were incubated at 37 °C for an additional 24 and 48 h, respectively.

Each morphological type of colonies was identified by the matrix-assisted laser desorption/ionization time-of-flight mass spectrometry (MALDI-TOF MS) (Microflex LT system; Bruker Daltonics, Kamnik, Slovenia) using MALDI Biotyper 3.1 software (Bruker Daltonics, Kamnik, Slovenia) according to the manufacturer’s instructions.

### 2.8. Statistical Analyses

The Shapiro–Wilk test was used to test the normal distribution of data. Since data were normally distributed, parametric tests were performed.

Statistical analysis was performed using R statistical software, version 3.5.2 (R Foundation for Statistical Computing, Vienna, Austria, 2018), and P 0.05 was considered significant.

Parametric *t*-test for independent samples were used to find the differences in relative growth rate between the puppies born via EL-CS, EM-CS and the puppies born via VD. The same test was used to test the relationship between the puppy’s growth rate among puppies born without the bacteria present in the meconium and puppies born with bacterial species present in the meconium.

## 3. Results

### 3.1. Collection of Samples

The collection of samples took place during December 2016 and July 2018. The dams were of eleven different breeds, and they were between 1.8–7 years old (mean age was 4.3 years) (Table 1). Seventy-nine puppies were born to 14 dams during 16 parturitions (two Boston Terriers whelped twice) (Table 2). There were three VDs, nine EL-CSs and four EM-CSs included in the study. The number and proportion (%) of puppies according to the type of parturition, gender and survival of newborns are presented in Table 2. Stillborn puppies (*n* = 2) were born by EM-CS and were excluded from the statistical analysis.

### 3.2. Bacteria Isolation

#### 3.2.1. Bacteria Isolated from Meconium and Colostrum

Meconium samples from two puppies were not obtained. The bacterial species isolated from the colostrum of the dams and the bacterial species isolated from the puppies’ meconium are presented in Table 3.

#### 3.2.2. Meconium Samples

The meconium samples were collected from 77 puppies (97.5%). The number of bacterial isolates from the meconium of the puppies are presented in Table 4.

From the meconium, 48 different bacterial species were isolated. They belonged to 24 different genera, mainly to *Staphylococcus* (22.92%), *Streptococcus* (16.67%), *Bacillus* (10.42%), *Pasteurella* (4.17%), *Haemophilus* (4.17%) *Kocuria* (4.17%), *Lactobacillus* (4.17%), *Cutibacterium* (4.17%), *Rothia* (4.17%), and *Acinetobacter* (4.17%). The most common bacterial species isolated from the meconium samples were *Staph. warneri* (32.5%), *Staph. epidermidis* (29.9%), *Staph. pseudintermedius* (15.6%), *C. acnes* (14.3%), *Staph. aureus* (3.9%) and *N. zoodegmatis* (3.9%).

#### 3.2.3. Bacteria Isolated from the Colostrum Samples According to the Type of Parturition

The colostrum samples were obtained from all of the dams (100%). At birth, no bacteria were isolated from 5/16 of the births (31.3%) and 4 h after the birth from 3/16 births (18.8%), all from CSs. No bacteria were isolated from 2 dams both from EL-CS. The bacterial species isolated from the colostrum according to the type of parturition are shown in Table 5.

Twenty-three different bacterial species belonging to 15 different genera were isolated from the colostrum samples. The bacterial colonies mainly belonged to the genera *Staphylococcus* (34.7%), *Kocuria* (8.7%) and *Lactobacillus lacti* (5%).

##### Relation between Colostrum Microbiota and the Type of Parturition

**Figure 1:**
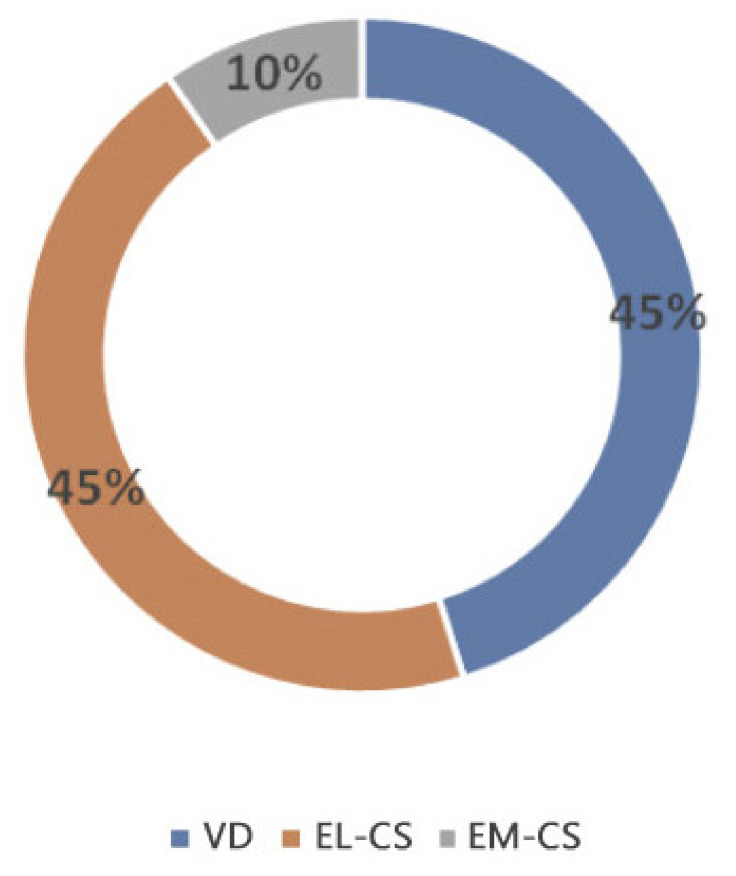
Bacterial richness of the colostrum isolates according to the type of parturition.

When comparing the microbiota composition of EL-CS, EM-CS and VD colostrum, several differences were found. In particular, the EL-CS and VD groups showed greater bacterial richness when compared with EM-CS group. Further, the EL-CS and VD groups were richer in microorganisms of the genera *Staphylococcus, Kocuria* and *Enterococcus* compared with EM-CS colostrum samples. Bacterial richness of the colostrum isolates according to the type of parturition is presented in Figure 1.

##### Prevalence of Bacteria in Colostrum Samples According to Their Oxygen Requirement

A greater number of anaerobic (63.2%) compared with aerobic (36.8%) bacteria were isolated in the colostrum of all three groups. Interestingly, VD colostrum showed a greater number of anaerobic bacterial genera (68.9%) compared with both CS groups (57.5%). Moreover, only a few anaerobic bacterial genera were isolated in the EM-CS group with low diversity of bacterial species. The VD group showed a higher abundance of intestinal bacterial genera (58.4%) compared with CS groups (49.3%). Environmental microorganisms were presented in all three groups, but CS colostrum (50.7%) had higher numbers of environmental bacteria compared with VD colostrum (41.6%).

### 3.3. Bacteria Isolated from the Colostrum and the Meconium Samples

The most prevalent bacteria isolated from dams’ colostrum belonged to genera *Staphylococcus*, *Kocuria* and *Enterococcus*, while the most prevalent bacteria genera isolated from the meconium samples were *Staphylococcus* (22.92%), *Streptococcus* (16.67%), *Bacillus* (10.42 %), *Pasteurella* (4.17%), *Haemophilus* (4.17%) *Kocuria* (4.17%), *Lactobacillus* (4.17%), *Cutibacterium* (4.17%), *Rothia* (4.17%) and *Acinetobacter* (4.17%). The correlation between the dam’s colostrum and the puppies’ meconium is presented in the Table 6.

#### Comparison between the Colostrum and the Meconium Microbiota According to the Type of Parturition

Puppies that were born vaginally all had the same bacterial isolates in their meconium as in their mother’s colostrum. However, in CS groups, we isolated the same bacteria species only in 53.8% of the births (in 55.56% of the EL-CS and in 25% of the EM-CS). The comparison of whether the same bacterial species were found in the puppies’ meconium as in the dam’s colostrum is shown in Table 6.

### 3.4. Growth Rate

On the first day, the difference between the groups was not significant, but puppies born via VD lost on average only 0.4% of their body weight, while those born via EM-CS lost on average 1.8%, and EL-CS 2.4% of the body weight. The puppies born via VD had the highest and the puppies born via EL-CS, had the lowest weight gain on days 1, 2, and 3 after birth. However, on day 4, the puppies born via VD still had the highest, while the puppies born via EM-CS had the lowest relative weight gain.

**Figure 2:**
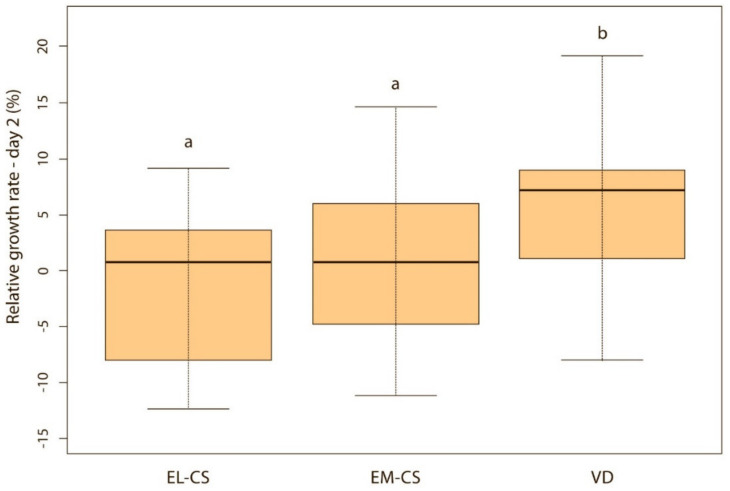
A comparison of relative weight increase on the 2nd day in puppies born with EL-CS, EM-CS and VD. Different superscript letters indicate significant difference (*p* < 0.01).

**Figure 3:**
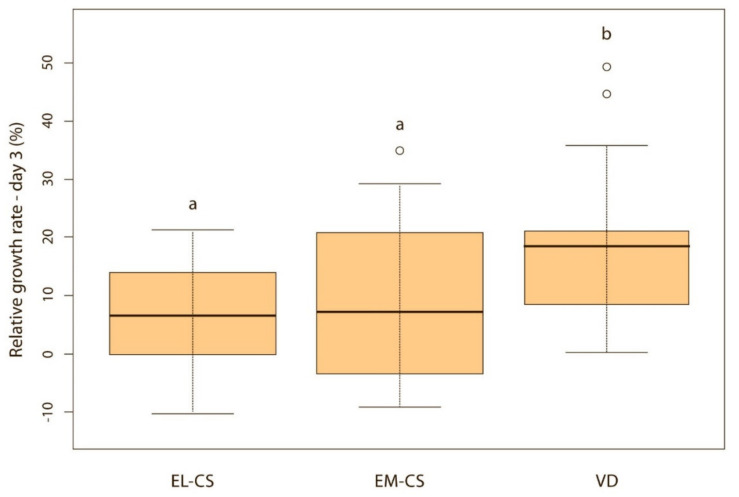
A comparison of relative weight gain in puppies born with EL-CS, EM-CS and VD on the 3rd day. Significant difference is marked with different superscripts (*p* < 0.05).

**Figure 4:**
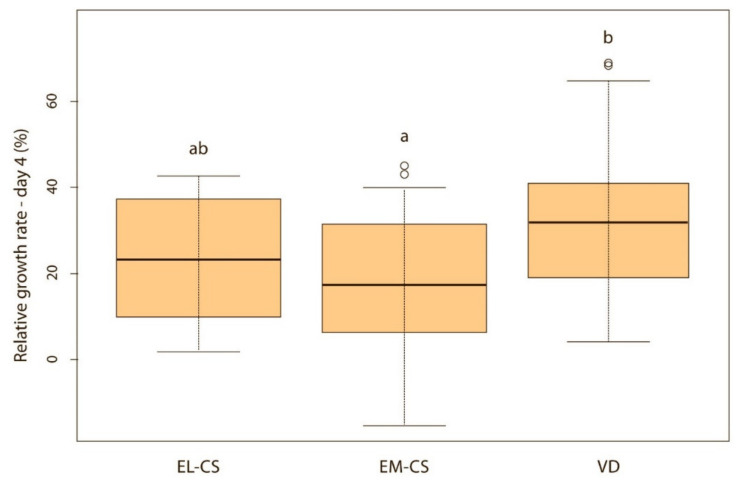
A comparison of relative weight gain in puppies born with EL-CS, EM-CS and VD on the 4th day. Significant difference is marked with different superscripts (*p* < 0.05).

Vaginally born puppies had a statistically significantly higher relative weight gain on the second (*p* < 0.01) (Figure 2) and third day (*p* < 0.05) (Figure 3) compared with the two CS groups. On the fourth day, the difference was statistically significant only between VD and EM-CS groups (*p* < 0.05) (Figure 4). Later on, until 8 weeks of age, no statistically significant difference in the relative weight gain was observed between puppies born with VD, EL-CS and EM-CS (*p* > 0.05).

#### The Correlation between the Relative Weight Gain between the Puppies That Had Bacteria Present in the Meconium and Those without Bacteria in the Meconium

**Figure 5:**
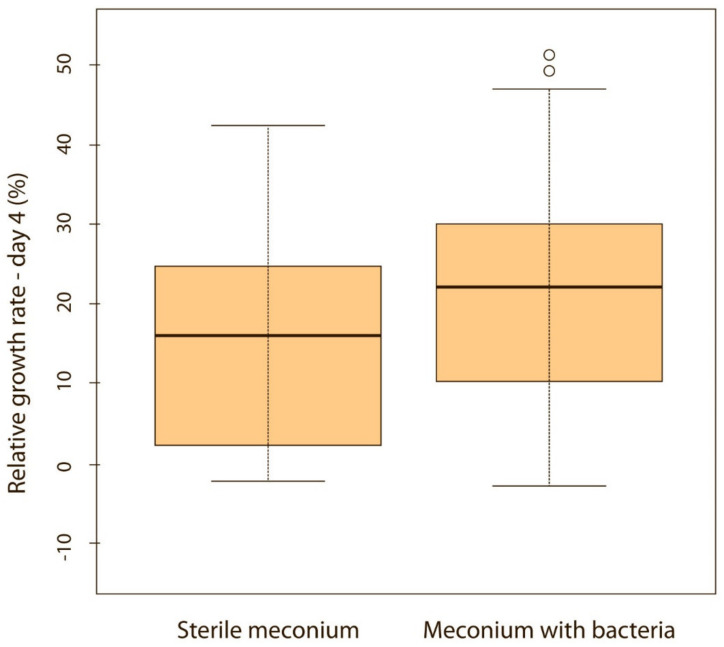
Relative weight gain between puppies with or without bacteria present in their meconium on the 4th day (*p* < 0.05). Note: Sterile meconium does not mean that there were no bacteria in the meconium. We were unable to isolate them with the technique used, and it is possible that choosing a different, more sensitive technique would yield different results.

**Figure 6:**
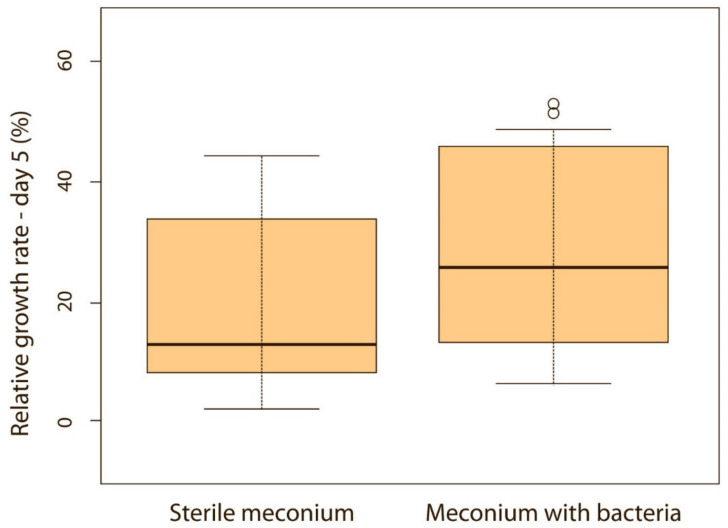
Relative weight gain between puppies with or without bacteria present in their meconium on the 5th day (*p* < 0.05). Note: Sterile meconium does not mean that there were no bacteria in the meconium. We were unable to isolate them with the technique used, and it is possible that choosing a different, more sensitive technique would yield different results.

When puppies were born without bacteria present in their meconium, their weight gain was lower compared with puppies that had bacteria present in the meconium. Although the difference was not significant on the first, second and third day of life (*p* > 0.05), it became significant on the fourth (*p* < 0.05) (Figure 5) and fifth day (*p* < 0.05) (Figure 6). Puppies’ weight gain was monitored until 8 weeks of life; later on, there was no significant difference in the weight gain between puppies with and without bacteria present in their meconium (*p* > 0.05).

## 4. Discussion

Colostrum can change its nutritional and microbiological composition quite rapidly and there are many factors, such as environment, stress and type of parturition, that can affect its composition [1]. Diet is also one of those factors and can have large impact, especially on the bacterial composition. For this reason, all dams that participated in the present study were fed a US Food and Drug Administration (FDA)-approved diet for pregnant dogs, which has very strict ingredient requirements for products containing at least 25% protein and 17% fat (FDA). No raw food was allowed.

Colostrum microbiota was shaped by the type of parturition in the present study since significant differences in isolated bacterial and in their prevalence were observed. Thus, the colostrum microbiota of VD and EL-CS puppies compared with EM-CS colostrum samples, was significantly richer in bacteria belonging to the genera *Staphylococcus spp, Lactobacillus, Kocuria* and *Enterococcus*. EM-CS had the highest abundance of *Haemophilus* and *Prevotella spp. Haemophilus* was more abundant in both CS groups but had a critical importance only in the EM-CS group. Interestingly, in humans, *Haemophilus* was more abundant in the colostrum of infants born vaginally. However, its presence was only important in the CS group, where it acted as one of the main bacteria. They suggested that this supports the thesis about bacterial pathogenicity which is not always related to the prevalence of bacteria present in the environment, but there are many other factors that can influence their pathogenicity, including the interactions with other microbes [9].

*Staphylococcus pseudintermedius* was one of the most abundant bacteria in the colostrum of VD and EL-CS group. In our previous study, *Staph. pseudintermedius* was also the major contributor of the oral and vaginal microbiota of dams [26]. Previously, *Staph. pseudintermedius* clones were found to be passed from the mother to the puppies around parturition [27]. In another study, *Staph. pseudintermedius* was isolated from puppies within 8 h after parturition in 78% of cases [28], and it was found to be a potential cause of sepsis in neonatal puppies [26]. However, it should be noted that *Staph. pseudintermedius* is part of the bacterial skin flora and a common opportunistic pathogen in dogs [29]. Rota et al. (2021), suggested that its isolation may therefore be due to contamination [30]. Even though we were very careful during sampling of the meconium in puppies (i.e., cleaning the anal area before sampling, sterile cotton swabs for sampling) and colostrum in the dam (i.e., disinfecting the skin before sampling, wearing sterile gloves and discarding first few drops of colostrum), bacterial contamination of samples cannot be completely ruled out. However, this bacterium was a major component of the colostrum microbiota (31.7%) in the present study and therefore, the possibility of contamination was reduced as much as is possible.

The high abundance of *Staphylococcus* and *Lactobacillus* in colostrum is logical, as these genera were found to be part of the human colostrum “core microbiota” and they are present in very high numbers in human milk of healthy women [31]. Since they are involved in lactate production and metabolism of lactate, their presence in the colostrum is considered as a part of healthy colostrum microbiota composition. As facultative anaerobes they are able to colonize the gastrointestinal tract early and, by using O_2_, they contribute to the formation of an anaerobic system environment for later colonization of strict anaerobes [32].

In our study, a greater number and a richer abundance of anaerobic bacteria were found. These results are in agreement with a study conducted in humans, where a higher number of anaerobic bacterial genera were found in colostrum compared with aerobic bacterial genera. However, the relative abundance of aerobic bacteria was also a major component of the colostrum microbiota [9], which is also consistent with our results where the presence of aerobic bacteria was found. Toscano et al. (2017) suggested that high oxygen content in colostrum could justify the greater abundance of aerobic bacteria [9]. In our study, the colostrum of all three groups contained at least some gut bacteria, which is consistent with the entero-mammary hypothesis that the colostrum microbiota is shaped by the gut bacteria present in the host. The difference in colostrum microbiota between the EL -CS and EM -CS groups is difficult to explain but could be due to the fact that the dams in the EL -CS group were healthy, whereas the dams in the EM -CS group are often exhausted, stressed, and at higher risk of anesthesia, which could contribute to the changes in the composition of their colostrum microbiota.

Several studies in women suggested that the breast milk microbiota plays a very important role in the establishment of the infant gut microbiome [6,32]. Studies on the vertical transmission of bacteria from ruminant milk to the offspring are sparse [16] and lacking in dogs. The origin of bacteria found in expressed milk probably originate from bacterial contamination during and between suckling, due to exposure of the mammary gland [33]. On the other hand, there is increasing evidence that the bacteria present in milk come through an endogenous route through the entero-mammary pathway, as has been studied in humans [34]. Recently, a study investigating the relationship between the colostrum microbiota and the early microbiome development of calves found that the colostrum microbiota has the same bacterial species as newborn calves, suggesting that colostrum contributes to the composition of the calf gastrointestinal microbiome [35]. A similar observation was made in our study, where it was found that the composition of the meconium microbiota of puppies present at birth was similar to the colostrum microbiota of their mothers’. The same bacterial species were found in all puppies born vaginally and in 53.8% of puppies delivered via CS.

The colostrum of mothers who underwent VD had a slightly greater bacterial richness compared with the EL-CS group. However, the VD and EL-CS groups both had much greater bacterial abundance than the EM-CS groups. Similar results were observed in a human study where the colostrum microbiota of VD infants was significantly richer in microorganisms compared with infants delivered with CS [9]. These results may also contribute to the higher weight gain we observed in puppies born via VD compared with both CS groups on the second and third day. On the fourth day, the difference in growth rate was statistically significant only between VD and EM-CS group, which had the lowest bacterial diversity. In our previous study, we also compared the growth rates of puppies born with VD compared with CS groups (elective and emergency cesarean section were categorized as CS group) and similar observations were found. Puppies born vaginally had a higher relative growth rate on days 2, 3, and 4 than puppies born with CS [28]. Bacterial diversity is important for maintaining and improving a stable and balanced environment. Therefore, animals that have higher biodiversity and greater richness of bacterial species are more resilient and adaptable to stress than those with only a few microorganisms [36]. This could influence the microbiome of puppies and contribute to their survival in the first days of life, when mortality rates are highest [37]. We can further confirm this with the finding that puppies born without the bacteria present in the meconium had a slower growth rate compared with puppies with established meconium microbiota at birth.

The results refer only to bacteria that we were able to cultivate under aerobic and anaerobic conditions on blood agar. We are aware that the results could be even more complete if the study was carried out with methods that could prove the presence of even those microorganisms that cannot be cultivated, which may be a subject of further research.

The present study indicate that the type of parturition plays an important role in modulating the colostrum microbiota. Differences in the maternal microbiome were found between all three groups, but the VD and EL-CS groups showed more similarities compared with the EM-CS group. In particular, the EL-CS and VD groups showed greater bacterial richness when compared with the EM-CS group and were richer in microorganisms of the genera *Staphylococcus, Kocuria* and *Enterococcus* compared with EM-CS colostrum samples. These results are important, especially in breeds where C-section is frequently performed, as proper birth planning and EL-CS could lead to greater survival of puppies in the first days of life and minimize the occurrence of the fading puppy syndrome due to inadequate microbiota in the colostrum of dams that underwent EM-CS. Future research should focus on establishing whether the colostrum microbiome is associated with health outcome of the neonatal puppies.

## Figures and Tables

**Table 1:** Number and proportion (%) of female and male puppies according to the different breeds included in the study.

Breed size	Breed	Number and Proportion (%) of Parturitions	Number and Proportion (%) of Puppies	Number and Proportion (%) of Female Puppies	Number and Proportion (%) of Male Puppies
Small breeds (≤10 kg)	Miniature Schnauzer	1 (6.25)	5 (6.3)	2 (2.6)	3 (3.8)
	Jack Russell Terrier	1 (6.25)	4 (5.1)	1 (1.3)	3 (3.8)
	Pomeranian	1 (6.25)	3 (3.8)	1 (1.3)	2 (2.6)
	Miniature Poodle	1 (6.25)	2 (2.6)	1 (1.3)	1 (1.3)
	Boston Terrier	4 (25.0)	16 (20.2)	11 (13.9)	5 (6.3)
Total		8 (50.0)	30 (38.0)	16 (20.26)	14 (17.7)
Medium-large breeds	Whippet	1 (6.25)	7 (8.9)	4 (5.1)	3 (3.8)
(10.1–25 kg)	Pembroke Welsh Corgi	2 (12.5)	10 (12.6)	3 (3.8)	7 (8.9)
	English Bulldog	1 (6.25)	4 (5.1)	2 (2.6)	2 (2.5)
	French Bulldog	2 (12.5)	13 (16.5)	5 (6.3)	8 (10.1)
Total		6 (37.5)	34 (43.0)	14 (17.7)	20 (25.3)
Large to giant breeds (>25.1 kg)	Greater Swiss Mountain Dog	1 (6.25)	6 (7.6)	1 (1.3)	5 (6.3)
	Newfoundland	1 (6.25)	9 (11.4)	4 (5.1)	5 (6.3)
Total		2 (12.5)	15 (19.0)	5 (6.3)	10 (12.7)

**Table 2:** Number and proportion (%) of puppies according to the type of parturition, gender and survival of newborns.

			Number of Puppies (*n*)	Proportion of Puppies [%]
Type of parturition	Vaginal delivery		18	22.8
	Cesarean section	Elective	41	51.9
		Emergency	20	25.3
Sex of the puppies	Female		35	44.3
	Male		44	55.7
Survival	Born alive		77	97.5
	Stillborn puppies		2	2.5

**Table 3:** Bacterial species isolated from the colostrum of the dams and meconium of the puppies.

Gram-Positive Bacteria	COL	MEC
*Staphylococcus*		
*Staph. epidermidis*	*+*	*+*
*Staph. pseudintermedius*	*+*	*+*
*Staph. hominis*	*+*	*+*
*Staph. aureus*		*+*
*Staph. haemolyticus*	*+*	*+*
*Staph. warneri*	*+*	*+*
*Staph. capitis*	*+*	*+*
*Staph. pettenkoferi*		*+*
*Staph. caprae*		*+*
*Staph. simulans*	*+*	
*Staph. felis*	*+*	
*Staphylococcus spp.*	*+*	*+*
*Streptococcus*		
*Strep. salivarius*		*+*
*Strep. oralis*		*+*
*Strep. pluranimalium*		*+*
*Strep. mitis*		*+*
*Strep. sanguinis*		*+*
*Strep. parasanguinis*		*+*
*Strep. pneumoniae*		*+*
*Streptococcus spp.*		*+*
*Enterococcus faecalis*	*+*	
*Actinomyces*		
*A. odontolyticum*		*+*
*A. weissii*	*+*	
*Corynebacterium spp.*		*+*
*Rothia* *R. dentocariosa* *R. mucilaginosa*		*+* *+*
*Lactobacillus*		
*L. johnsonii*		*+*
*Lactobacillus lacti*	*+*	*+*
*Lactobacillus spp.*	*+*	*+*
*Macrococcus caseolyticus*		*+*
*Cutibacterium (Propionibacterium)*		
*C. acnes*	*+*	*+*
*C. granulosum*		*+*
*Bacillus*		
*B. flexus*		*+*
*B. megaterium*		*+*
*B. pumilus*		*+*
*B. simplex*		*+*
*Bacillus spp.*	*+*	*+*
*Kocuria*		
*K. kristinae*		*+*
*K. palustris*	*+*	
*Kocuria spp.*	*+*	*+*
*Streptomyces spp.*		*+*
*Macrococcus*		
*M. caseolyticus*		*+*
*Macrococcus spp.*	*+*	
**Gram-Negative Bacteria**	**COL**	**MEC**
*Neisseria*		
*N. weaveri*	*+*	
*N. zoodegmatis*		*+*
*Pasteurella*		
*P. canis*		*+*
*P. stomatis*		*+*
*Pasteurella spp.*	*+*	
*Haemophilus*		
*H. haemoglobinophilus*	*+*	*+*
*Haemophilus spp.*		*+*
*Moraxella*		
*M. osloensis*	*+*	
*Moraxella spp.*		*+*
*Acinetobacter*		
*A. schindleri*	*+*	
*A. junni*		*+*
*A. lwoffii*		*+*
*Klebsiella pneumoniae*	*+*	
*Paracoccus spp.*		*+*
*Escherichia coli*		*+*
*Pantoea spp.*	*+*	
*Brevundimonas aurantiaca*		*+*

Legend: COL, colostrum; MEC, meconium.

**Table 4:** Bacterial isolates from the meconium of the puppies according to the type of parturition.

Gram-Positive Bacteria	TOP	
*Staphylococcus*		
*Staph. epidermidis*	VD, EL-CS, EM-CS	
*Staph. pseudintermdius*	VD, EL-CS	
*Staph. hominis*	VD, EL-CS	
*Staph. aureus*	VD, EL-CS	
*Staph. haemolyticus*	VD, EL-CS	
*Staph. warneri*	VD, EL-CS, EM-CS	
*Staph. hominis*	VD, EL-CS	
*Staph. capitis*	EL-CS, EM-CS	
*Staph. pettenkoferi*	EL-CS	
*Staph. caprae*	EL-CS	
*Staphylococcus spp.*	VD, EL-CS, EM-CS	
*Streptococcus*		
*Strep. salivarius*	EL-CS	
*Strep. oralis*	EL-CS, EM-CS	
*Strep. pluranimalium*	EM-CS	
*Strep. mitis*	VD	
*Strep. sanguinis*	EM-CS, EL-CS	
*Strep. parasanguinis*	EL-CS	
*Strep. pneumoniae*	EL-CS	
*Streptococcus spp.*	VD	
*Actinomyces odontolyticum*	EL-CS	
*Corynebacterium spp.*	VD	
*Rothia*		
*R. dentocariosa*	EL-CS	
*R. mucilaginosa*	EM-CS	
*Lactobacillus*		
*L. johnsonii*	VD, EL-CS	
*L. lacti*	VD, EL-CS	
*Lactobacillus spp.*	EL-CS	
*Bacillus*		
*B. flexus*	VD	
*B. megaterium*	VD	
*B. pumilus*	VD	
*B. simplex*	EM-CS	
*Bacillus spp.*	VD	
*Kocuria*		
*K. kristinae*	EL-CS	
*Kocuria spp.*	VD	
*Macrococcus caseolyticus*	VD	
*Streptomyces spp*	VD	
*Cutibacterium*		
*C. acne*	EL-CS, VD, EM-CS	
*C. granulosum*	EM-CS	
**Gram-Negative Bacteria**	**COL**	**MEC**
*Neisseria*		
*N. weaveri*	*+*	
*N. zoodegmatis*		*+*
*Pasteurella*		
*P. canis*		*+*
*P. stomatis*		*+*
*Pasteurella spp.*	*+*	
*Haemophilus*		
*H. haemoglobinophilus*	*+*	*+*
*Haemophilus spp.*		*+*
*Moraxella*		
*M. osloensis*	*+*	
*Moraxella spp.*		*+*
*Acinetobacter*		
*A. schindleri*	*+*	
*A. junni*		*+*
*A. lwoffii*		*+*
*Klebsiella pneumoniae*	*+*	
*Paracoccus spp.*		*+*
*Escherichia coli*		*+*
*Pantoea spp.*	*+*	
*Brevundimonas aurantiaca*		*+*

Legend: COL—colostrum, MEC—meconium.

**Table 5:** Bacterial isolates from the colostrum according to the type of parturition.

Gram-Positive Bacteria	Type of Parturition	Gram-Negative Bacteria	Type of Parturition
*Staphylococcus*		*Neisseria weaveri*	VD
*Staph. epidermidis*	EL-CS		
*Staph. pseudintermedius*	EL-CS, VD		
*Staph. haemolyticus*	EL-CS		
*Staph. warneri*	EL-CS		
*Staph. hominis*	EL-CS		
*Staph. capitis*	EL-CS		
*Staph. pettenkoferi*	EL-CS		
*Staph. simulans*	EL-CS, VD		
*Enterococcus faecalis*	VD, EL-CS	*Pasteurella spp.*	VD
*Actinomyces meissii*	EL-CS	*Haemophilus haemoglobinophilus*	VD
*Lactobacillus spp.*	VD	*Moraxella osloensis*	EM-CS
*Cutibacterium acnes*	EL-CS, EM-CS	*Acinetobacter schindleri*	VD
*Bacillus cereus*	EM-CS	*Klebsiella pneumoniae*	VD
*Kocuria*		*Panthoea spp.*	VD
*K. palustris*	EL-CS		
*Kocuria spp.*	EL-CS, VD		
*Macrococcus spp.*	VD		

**Table 6:** Comparison of whether the same bacterial species were isolated from the puppies’ meconium and the dam’s colostrum.

	Vaginal Delivery			Cesarean Section												
				EL-CS									EM-CS			
N	1	2	3	4	5	6	7	8	9	10	11	12	13	14	15	16
C→M	Y	Y	Y	Y	N	N	N	Y	N	Y	Y	Y	N	Y	N	N

Legend: N = parturition number, C = colostrum, M = meconium, Y = the same bacterial species were isolated from the colostrum and meconium, N = the same bacterial species were not isolated from the colostrum and meconium.

## Data Availability

Data are available from the authors upon request.

## References

[1] Drago L., Toscano M., De Grandi R., Grossi E., Padovani E.M., Peroni D.G. (2017). Microbiota network and mathematic microbe mutualism in colostrum and mature milk collected in two different geographic areas: Italy versus Burundi. ISME J..

[2] Borghesi J., Mario L., Rodrigues M., Favaron P., Miglino M. (2014). Immunoglobulin Transport during Gestation in Domestic Animals and Humans—A Review. Open J. Anim. Sci..

[3] Chastant-Maillard S., Aggouni C., Albaret A., Fournier A., Mila H. (2017). Canine and feline colostrum. Reprod. Domest. Anim..

[4] Satyaraj E., Reynolds A., Pelker R., Labuda J., Zhang P., Sun P. (2013). Supplementation of diets with bovine colostrum influences immune function in dogs. Br. J. Nutr..

[5] Gopalakrishna K.P., Hand T.W. (2020). Influence of Maternal Milk on the Neonatal Intestinal Microbiome. Nutrients.

[6] Fernández L., Langa S., Martín V., Maldonado A., Jiménez E., Martín R., Rodríguez J.M. (2013). The human milk microbiota: Origin and potential roles in health and disease. Pharmacol. Res..

[7] Fitzstevens J.L., Smith K.C., Hagadorn J.I., Caimano M.J., Matson A.P., Brownell E.A. (2016). Systematic review of the human milk microbiota. Nutr. Clin. Pract..

[8] Khodayar-Pardo P., Mira-Pascual L., Collado M.C., Martínez-Costa C. (2014). Impact of lactation stage, gestational age and mode of delivery on breast milk microbiota. J. Perinatol..

[9] Toscano M., De Grandi R., Peroni D.G., Grossi E., Facchin V., Comberiati P., Drago L. (2017). Impact of delivery mode on the colostrum microbiota composition. BMC Microbiol..

[10] Holmlund U., Amoudruz P., Johansson M.A., Haileselassie Y., Ongoiba A., Kayentao K., Traore B., Doumbo S., Schollin J., Doumbo O. (2010). Maternal country of origin, breast milk characteristicsand potential influences on immunity in offspring. Clin. Exp. Immunol..

[11] Amoudruz P., Holmlund U., Schollin J., Sverremark-Ekström E., Montgomery S.M. (2009). Maternal country of birth and previous pregnancies are associated with breastmilk characteristics. Pediatr. Allergy Immunol..

[12] Sohn K., Kalanetra K.M., Mills D.A., Underwood M.A. (2016). Buccal administration of human colostrum: Impact on the oral microbiota of premature infants. J. Perinatol..

[13] Indrebø A., Trangerud C., Moe L. (2007). Canine neonatal mortality in four large breeds. Acta Vet. Scand..

[14] Mila H., Grellet A., Chastant-Maillard S. Prognostic value of birth weight and early weight gain on neonatal and pediatric mortality: A longitudinal study on 870 puppies. Proceedings of the Program and Presented at the 7th International Symposium on Canine and Feline Reproduction 2012.

[15] Hunt K.M., Foster J.A., Forney L.J., Schütte U.M., Beck D.L., Abdo Z., Fox L.K., Williams J.E., McGuire M.K., McGuire M.A. (2011). Characterization of the diversity and temporal stability of bacterial communities in human milk. PLoS ONE.

[16] Addis M.F., Tanca A., Uzzau S., Oikonomou G., Bicalho R.C., Moroni P. (2016). The bovine milk microbiota: Insights and perspectives from -omics studies. Mol. Biosyst..

[17] Falentin H., Rault L., Nicolas A., Bouchard D.S., Lassalas J., Lamberton P., Aubry J.M., Marnet P.G., Le Loir Y., Even S. (2016). Bovine teat microbiome analysis revealed reduced alpha diversity and significant changes in taxonomic profiles in quarters with a history of mastitis. Front. Microbiol..

[18] Quigley L., O’Sullivan O., Stanton C., Beresford T.P., Ross R.P., Fitzgerald G.F., Cotter P.D. (2013). The complex microbiota of raw milk. FEMS Microbiol. Rev..

[19] McInnis E.A., Kalanetra K.M., Mills D.A., Maga E.A. (2015). Analysis of raw goat milk microbiota: Impact of stage of lactation and lysozyme on microbial diversity. Food Microbiol..

[20] Treven P., Mrak V., Bogović Matijašić B., Horvat S., Rogelj I. (2015). Administration of probiotics Lactobacillus rhamnosus GG and Lactobacillus gasseri K7 during pregnancy and lactation changes mouse mesenteric lymph nodes and mammary gland microbiota. J. Dairy Sci..

[21] Catozzi C., Sanchez Bonastre A., Francino O., Lecchi C., De Carlo E., Vecchio D., Martucciello A., Fraulo P., Bronzo V., Cuscó A. (2017). The microbiota of water buffalo milk during mastitis. PLoS ONE.

[22] Li Z., Wright A.-D.G., Yang Y., Si H., Li G. (2017). Unique bacteria community composition and co-occurrence in the milk of different ruminants. Sci. Rep..

[23] Soto Del Rio M.L.D., Dalmasso A., Civera T., Bottero M.T. (2017). Characterization of bacterial communities of donkey milk by high-throughput sequencing. Int. J. Food Microbiol..

[24] Aakko J., Kumar H., Rautava S., Wise A., Autran C., Bode L., Isolauri E., Salminen S. (2017). Human milk oligosaccharide categories define the microbiota composition in human colostrum. Benef. Microbes.

[25] Derakhshani H., Plaizier J.C., De Buck J., Barkema H.W., Khafipour E. (2018). Composition of the teat canal and intramammary microbiota of dairy cows subjected to antimicrobial dry cow therapy and internal teat sealant. J. Dairy Sci..

[26] Zakošek Pipan M., Švara T., Zdovc I., Papić B., Avberšek J., Kušar D., Mrkun J. (2019). *Staphylococcus pseudintermedius* septicemia in puppies after elective cesarean section: Confirmed transmission via dam’s milk. BMC Vet. Res..

[27] Paul M., Bishara J., Yahav D., Goldberg E., Neuberger A., Ghanem-Zoubi N., Dickstein Y., Nseir W., Dan M., Leibovici L. (2015). Trimethoprim-sulfamethoxazole versus vancomycin for severe infections caused by meticillin resistant Staphylococcus aureus: Randomised controlled trial. BMJ.

[28] Saijonmaa-Koulumies L.E., Lloyd D.H. (2002). Colonization of neonatal puppies by Staphylococcus intermedius. Vet. Dermatol..

[29] Bannoehr J., Guardabassi L. (2012). Staphylococcus pseudintermediusin the dog: Taxonomy, diagnostics, ecology, epidemiology and pathogenicity. Vet. Dermatol..

[30] Rota A., Del Carro A., Bertero A., Del Carro A., Starvaggi Cucuzza A., Banchi P., Corrò M. (2021). Does Bacteria Colonization of Canine Newborns Start in the Uterus?. Animals.

[31] Kumar H., Toit E., Kulkarni A., Aakko J., Linderborg K.M., Zhang Y., Nicol M.P., Isolauri E., Yang B., Collado M.C. (2016). Distinct patterns in human milk microbiota and fatty acid profiles across specific geographic locations. Front. Microbiol..

[32] Cerdó T., Ruiz A., Acuña I., Jáuregui R., Jehmlich N., Haange S.B., von Bergen M., Suárez A., Campoy C. (2018). Gut microbial functional maturation and succession during human early life. Environ. Microbiol..

[33] Rautava S., Luoto R., Salminen S., Isolauri E. (2012). Microbial contact during pregnancy, intestinal colonization and human disease. Nat. Rev. Gastroenterol. Hepatol..

[34] Williams J.E., Carrothers J.M., Lackey K.A., Beatty N.F., York M.A., Brooker S.L., Shafii B., Price W.J., Settles M.L., McGuire M.A. (2017). Human milk microbial community structure is relatively stable and related to variations in macronutrient and micronutrient intakes in healthy lactating women. J. Nutr..

[35] Yeoman C.J., Ishaq S.L., Bichi E., Olivo S.K., Lowe J., Aldridge B.M. (2018). Biogeographical differences in the influence of maternal microbial sources on the early successional development of the bovine neonatal gastrointestinal tract. Sci. Rep..

[36] Lozupone C.A., Stombaugh J.I., Gordon J.I., Jansson J.K., Knight R. (2012). Diversity, stability and resilience of the human gut microbiota. Nature.

[37] Tønnessen R., Borge K.S., Nødtvedt A., Indrebø A. (2012). Canine perinatal mortality: A cohort study of 224 breeds. Theriogenology.

